# Automated analysis of vessel morphometry in retinal images from a Danish high street optician setting

**DOI:** 10.1371/journal.pone.0290278

**Published:** 2023-08-24

**Authors:** Josefine Freiberg, Roshan A. Welikala, Jens Rovelt, Christopher G. Owen, Alicja R. Rudnicka, Miriam Kolko, Sarah A. Barman, on behalf of the FOREVER consortium

**Affiliations:** 1 Department of Drug Design and Pharmacology, University of Copenhagen, Copenhagen, Denmark; 2 School of Computer Science and Mathematics, Kingston University, Surrey, United Kingdom; 3 Population Health Research Institute, St. George’s, University of London, London, United Kingdom; 4 Department of Ophthalmology, Copenhagen University Hospital, Rigshospitalet, Glostrup, Copenhagen, Denmark; Federal University of Rio de Janeiro: Universidade Federal do Rio de Janeiro, BRAZIL

## Abstract

**Purpose:**

To evaluate the test performance of the QUARTZ (QUantitative Analysis of Retinal vessel Topology and siZe) software in detecting retinal features from retinal images captured by health care professionals in a Danish high street optician chain, compared with test performance from other large population studies (i.e., UK Biobank) where retinal images were captured by non-experts.

**Method:**

The dataset FOREVERP (Finding Ophthalmic Risk and Evaluating the Value of Eye exams and their predictive Reliability, Pilot) contains retinal images obtained from a Danish high street optician chain. The QUARTZ algorithm utilizes both image processing and machine learning methods to determine retinal image quality, vessel segmentation, vessel width, vessel classification (arterioles or venules), and optic disc localization. Outcomes were evaluated by metrics including sensitivity, specificity, and accuracy and compared to human expert ground truths.

**Results:**

QUARTZ’s performance was evaluated on a subset of 3,682 images from the FOREVERP database. 80.55% of the FOREVERP images were labelled as being of adequate quality compared to 71.53% of UK Biobank images, with a vessel segmentation sensitivity of 74.64% and specificity of 98.41% (FOREVERP) compared with a sensitivity of 69.12% and specificity of 98.88% (UK Biobank). The mean (± standard deviation) vessel width of the ground truth was 16.21 (4.73) pixels compared to that predicted by QUARTZ of 17.01 (4.49) pixels, resulting in a difference of -0.8 (1.96) pixels. The differences were stable across a range of vessels. The detection rate for optic disc localisation was similar for the two datasets.

**Conclusion:**

QUARTZ showed high performance when evaluated on the FOREVERP dataset, and demonstrated robustness across datasets, providing validity to direct comparisons and pooling of retinal feature measures across data sources.

## Introduction

The retina of the eye is considered a part of the central nervous system (CNS) and is said to be a window to the brain and circulatory system [1,2]. Not only do the retina and brain share anatomical and embryonic development characteristics, but also the microvascular circulation and regulation in the brain and retina are similar [1]. Viewing the vessels of the retina provides a unique opportunity to study the blood circulatory system. While systemic blood circulation can be visualized by using invasive procedures such as angiography x-ray examinations, the vessels of the retina are captured by non-invasive fundus images. Changes in retinal vessel tortuosity and diameter have previously been linked to cardiovascular disease, diabetes, and glaucoma [3–5]. Also, CNS and systemic diseases such as ischemic brain incidence, stroke, multiple sclerosis, and Alzheimer’s disease have recognized ocular manifestations [2]. Thus, it is evident to believe, that retinal vessels may be a biomarker for identifying early signs of both ocular and systemic diseases and can be used as a predictor for disease development.

Retinal imaging is part of a routine eye examination when visiting an ophthalmologist. In recent years it has gained popularity in high street optician chains, as retinal imaging demands limited training and captures important signs of disease pathology such as changes in the optic nerve, macula and blood vessels of the retina [6]. The interest in using artificial intelligence (AI) in healthcare as a supplement to routine eye examinations is growing, as its ability to help clinicians manage routine tasks and analyse large amounts of data effectively has the potential to transform healthcare [7–10]. Image recognition/diagnosis classification and the search for new prognostic risk factors are of particular interest [11]. Automation and AI in ophthalmology spans a multitude of approaches, from traditional image processing (unsupervised techniques e.g., edge detection or morphological operators), to machine learning techniques (e.g., supervised learning and unsupervised learning), which use hand-crafted features, to deep learning (a subfield of machine learning) that can automatically learn features. Current research in AI software for automatic analysis of retinal fundus images includes studies on cardiovascular disease, diabetic retinopathy, age-related macular degeneration, retinopathy of prematurity, neonatal fundus haemorrhages, glaucoma, and retinal breaks and detachments [11–21].

Vessel morphometry (also known as vasculometry) is an approach for studying biomarkers of disease. This requires retinal images to be converted into quantitative measurements, including measures of vessel width, area, and tortuosity. However, this is a time-consuming task for human observers and not feasible for studies examining vasculometric associations with disease, which demand big sample sizes to generate enough power to study small, yet meaningful group differences [22]. Hence, different software programmes for automated vessel analysis have been developed [23,24], including QUARTZ (QUantitative Analysis of Retinal vessel Topology and siZe) [25].

**Fig 1:**
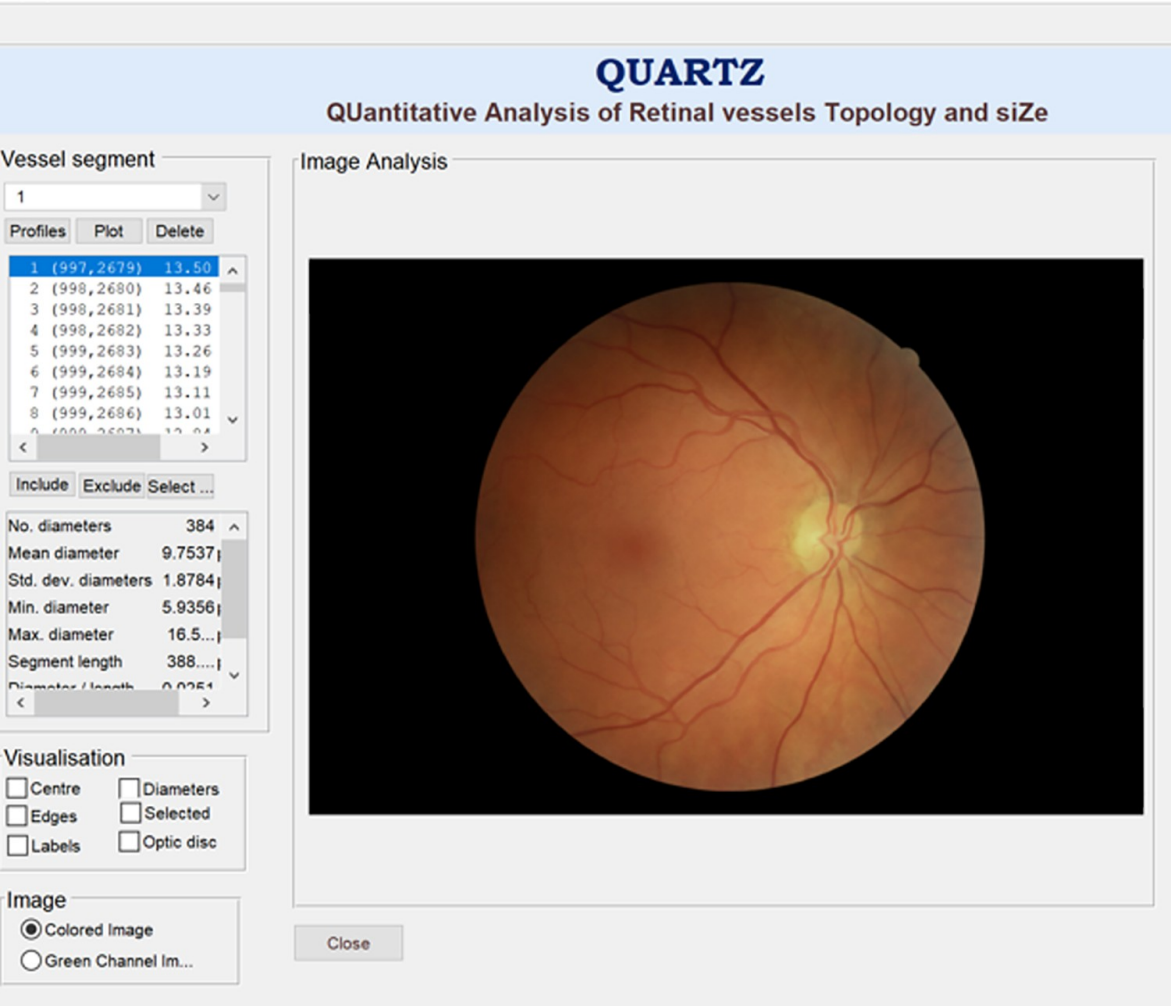
QUARTZ interface.

QUARTZ converts retinal images into quantitative measures of vessel morphometry for use in epidemiological studies [26–28]. QUARTZ analyses the entire retina (not limited to concentric areas around the optic disc), and evaluates image quality, vessel segmentation, arteriole/venule (A/V) classification, width, area, and tortuosity measurements of retinal vessels, and localisation of the optic disc (Fig 1) [4].

QUARTZ has previously been validated on the UK Biobank dataset [27]. The UK Biobank contains data from more than 502,656 UK citizens (40–69 years of age) collected from 2006 to 2010 [29]. Of all the participants, 68,549 had retinal images (45-degree field-of-view and 2048 x 1536 pixels image size) taken at baseline [27,29]. Output data from QUARTZ has previously been combined with epidemiological data from the UK Biobank cohort in the search for potential new biomarkers of disease. Studies have investigated the associations between vessel morphometry and glaucoma as well as cardiometabolic risk factors, including its ability to predict myocardial infarction and stroke [4,30–33]. Although QUARTZ was originally developed for use in UK Biobank, it is relevant to examine the performance of QUARTZ on multiple datasets using different image capture systems with images taken by experts and non-experts, as future versions of QUARTZ may be targeted at the clinic and hence should demonstrate robustness and high performance with few limitations across datasets [7,34]. Thus, the aim of this paper was to validate QUARTZ on a further dataset with a different image acquisition protocol. The performance of QUARTZ was investigated on the FOREVERP (Finding Ophthalmic Risk and Evaluating the Value of Eye exams and their predictive Reliability, Pilot) dataset from a Danish optician chain, and QUARTZ’s generalizability across datasets was examined by comparing the performance with previously published data from the UK Biobank.

## Methods

The methods applied in this paper have been detailed previously by Welikala et al. [27]. The same methods were used to ensure the comparability of performance parameters across datasets.

### The FOREVER dataset

Project FOREVER has been approved by the National Committee on Health Research Ethics, Denmark (project id H-21026000). The design and methodology of project FOREVER has been described thoroughly by Freiberg. et al. [35]. For participants enrolling in project FOREVER, informed written consent will be collected. The FOREVER (Finding Ophthalmic Risk and Evaluating the Value of Eye exams and their predictive Reliability) dataset contains data from Danish citizens, aged above 18 years, visiting an optician shop in Denmark. The dataset includes eye examinations: visual acuity, refraction, corneal thickness, intraocular pressure, retinal images and perimetries. A subset of the FOREVER dataset contains additional data on blood pressure, saliva samples for genetic analysis and Optical Coherence Tomography (OCT) scans. As Danish citizens have a unique social security number, the FOREVER dataset can be linked to the national registries enabling comprehensive linkage to disease risk and outcome data.

Enrolment of participants in the FOREVER cohort began in July 2022. The dataset used for validating QUARTZ to the FOREVER dataset consisted of images from the same Danish optician shops as in the FOREVER cohort. The dataset consisted of a subset of 3,682 images from 1,139 anonymized customers visiting an optician shop between February 2018 to May 2021. The dataset is referred to as “FOREVERP” (FOREVER, Pilot). The images were randomly selected for validation of QUARTZ and were not images from the FOREVER cohort, as validation was performed prior to the enrolment of participants in the FOREVER cohort. However, images from the FOREVERP dataset are comparable to images from the FOREVER dataset given that the image acquisition protocol is the same. Images from the FOREVERP can eventually be part of the FOREVER database if FOREVERP participants decide to enrol in the FOREVER cohort by given written consent.

The macular-centred retinal fundus images in “FOREVERP”were captured without mydriasis using digital non-mydriatic retinal cameras (Canon CR-2 AF) which incorporate Canon EOS 70D and Canon EOS 80D cameras. The retinal image photographers were trained personnel who either 1) attended a two-day course in fundus imaging, tonometry and perimetry enabling them to recognize errors and artefacts, or 2) had been trained by an optometrist. The optometrists are continuously trained with two mandatory and four optional training days per year, with a focus on specialized training in eye diseases such as glaucoma and diabetic retinopathy. The images were collected from multiple visits over several years, and the number of images varied per participant. Images were macular-centred and had a 45-degree field of view. Images were in BMP format and of multiple image sizes ranging from 1824 x 1216 pixels to 3984 x 2656 pixels, resized to 3984 x 2656 pixels.

### Performance parameters

The performance of the algorithm was compared with a reference standard or ground truth (GT). The GT was derived from data annotation performed by human observers (JF and RAW) [36] using purpose-built software. The performance of the algorithms was compared with the GT (e.g. comparison with labelled pixels, images, vessel segments, vessel widths etc.) and most were assessed by calculating the performance parameters of sensitivity, specificity and accuracy (Table 1) [37]. Sensitivity refers to the percentage of the positives that are correctly classified as positive (TP). Specificity refers to the percentage of negatives that are correctly classified as negative (TN). Accuracy refers to the proportion of the outcomes correctly predicted as either positive (TP) or negative (TN) [37,38].

**Table 1:** Performance parameters used for evaluation performance of algorithms; Sensitivity, specificity, and accuracy, TP = True Positive, TN = True Negative, FP = False Positive, and FN = False Negative.

Performance Parameter	Equation
Sensitivity	$\frac{TP}{\left(TP+FN\right)}$
Specificity	$\frac{TN}{\left(TN+FP\right)}$
Accuracy	$\frac{TP+TN}{\left(TP+TN+FP+TN\right)}$

### Automated image quality

**Fig 2:**
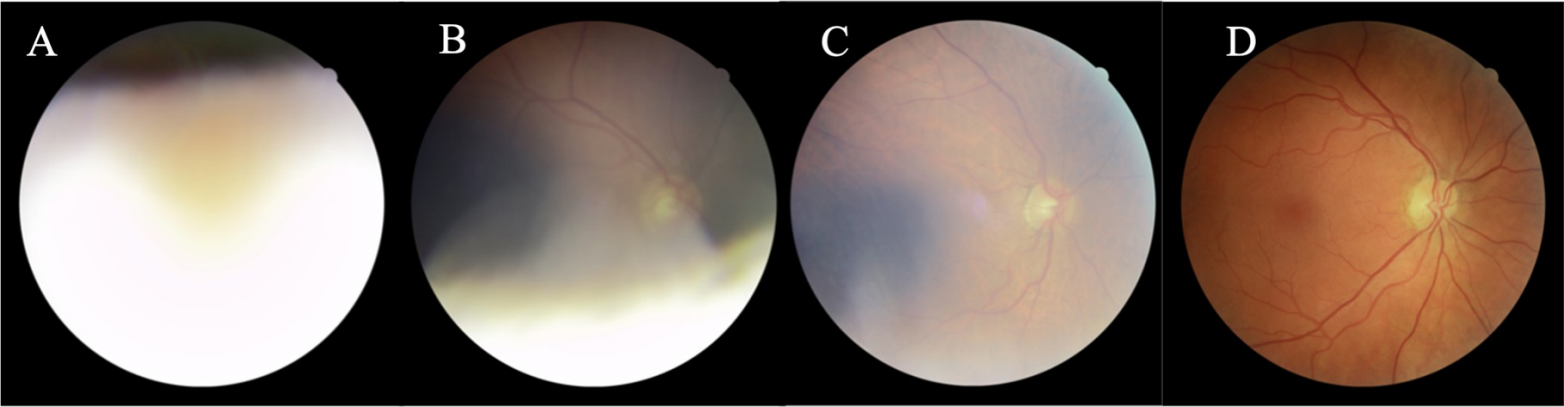
Automated image quality assessment of FOREVERP images performed by QUARTZ. **(**A and B) Examples of images of inadequate quality. (C and D) Examples of images of adequate quality.

Supervised learning (support vector machine classifier with the radial basis function kernel) along with global shape features (area, fragmentation, and complexity) measuring the segmented vessel map was used to classify images as either of inadequate or adequate quality. This approach was designed for use in epidemiological studies; hence an image can still be deemed adequate even if only a portion of the vasculature is visible [26] (Fig 2). 1,000 images were randomly selected and manually labelled by one human observer (RAW). Of the images, 826 were manually labelled as of adequate quality and 174 images as inadequate. The supervised classifier was trained with 500 images (using 5-fold cross-validation for model selection) and evaluated using a test data set of 500 images. A TP outcome equalled an image correctly classified as being of inadequate quality (Fig 2). The probability output from the classifier was normalized on a scale from 0 to 1 and flipped, to generate an image quality score (1 = highest quality).

### Vessel segmentation

**Fig 3:**
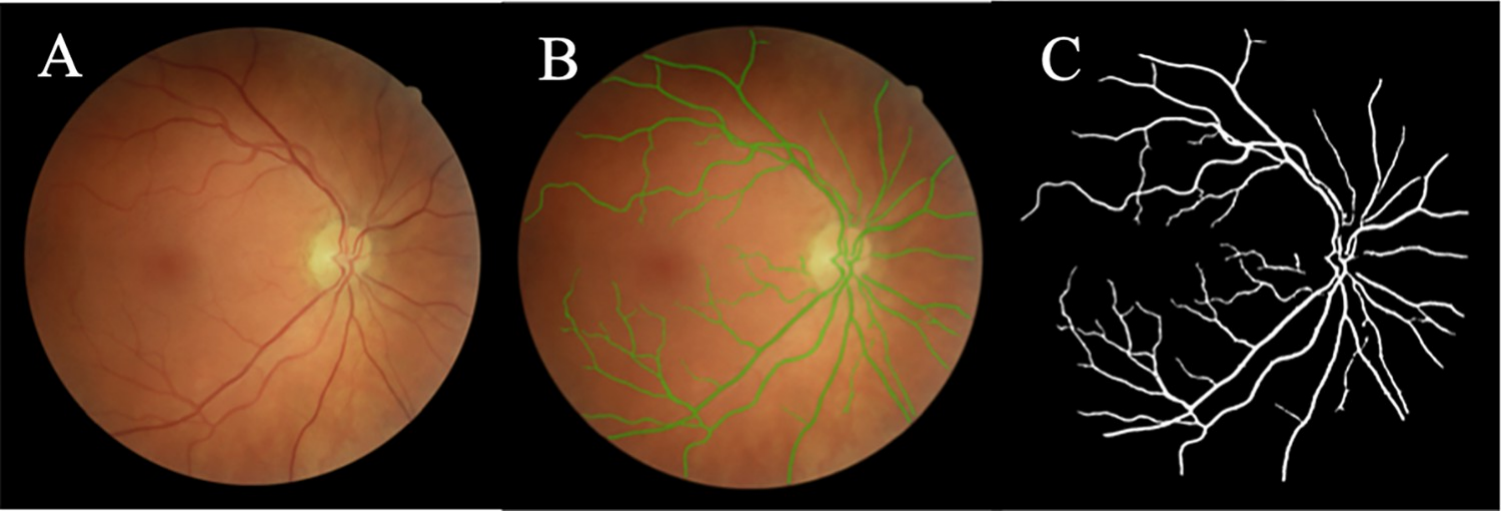
Automatic vessel segmentation of FOREVERP images performed by QUARTZ. (A) Retinal fundus images. (B) Segmentation of vessels (green). (C) Segmentation of vessels (white).

An unsupervised approach based on a multi-scale line detector and hysteresis thresholding based morphological reconstruction was used for vessel segmentation (Fig 3) [26]. The test set consisted of 10 randomly selected images of adequate quality. Two human observers (JF, RAW) manually labelled the test set independently, creating two separate sets of 10 images. The vessel segmentation from the first human observer (RAW) constituted the GT. The segmentations made by the second human observer (JF) were considered the target performance level that the automated segmentation should aim to achieve. The performance was evaluated per pixel with and without pre- and post-processing. Pre-processing refers to the removal of pixels of bright intensities whereas post-processing refers to the removal of the fovea and small objects falsely segmented as vessels [26,27].

### Vessel width measurements

**Fig 4:**
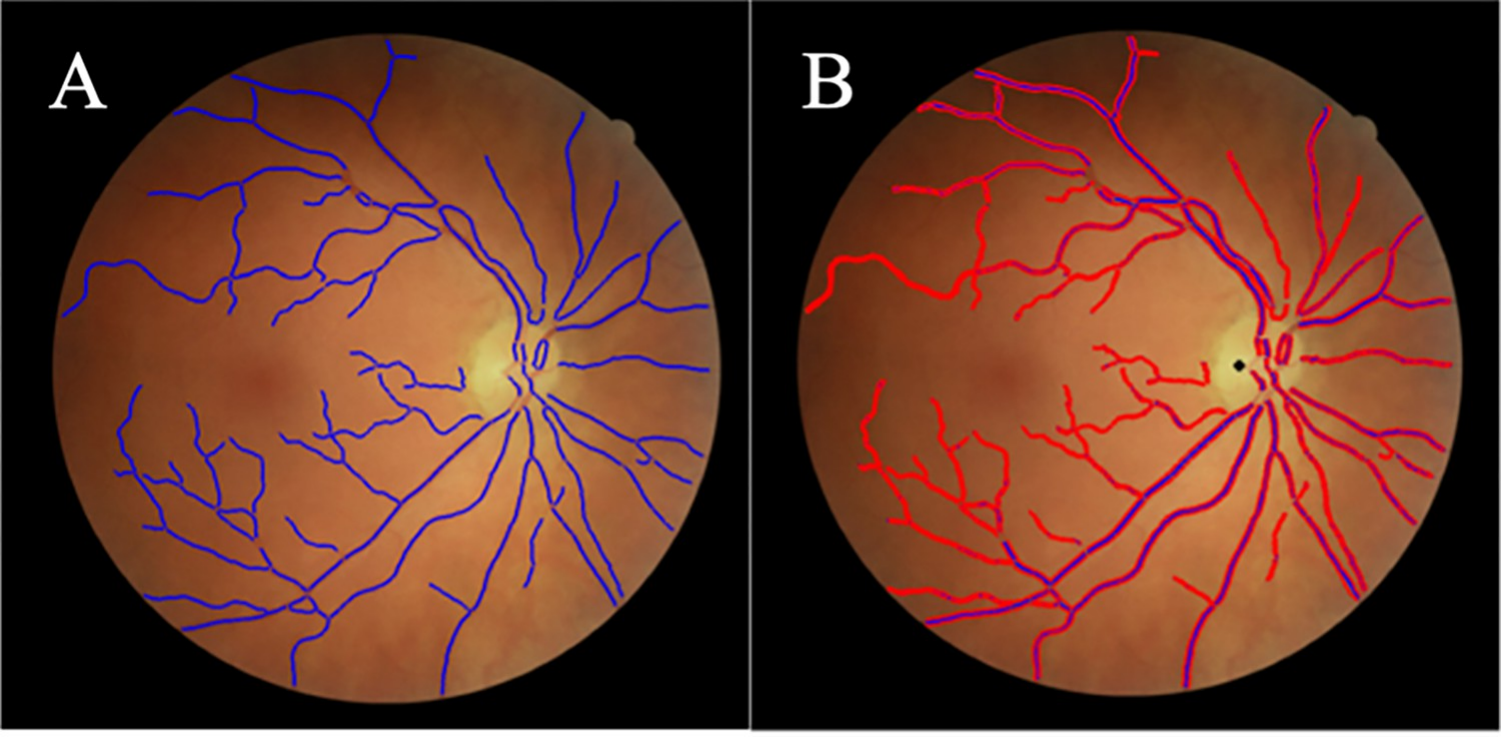
Automatic vessel width measurements performed by QUARTZ on FOREVERP images. (A) Vessel centre lines (blue). (B) Vessel centre lines (blue), vessel edges (red), and marking of optic disc (black).

**Fig 5:**
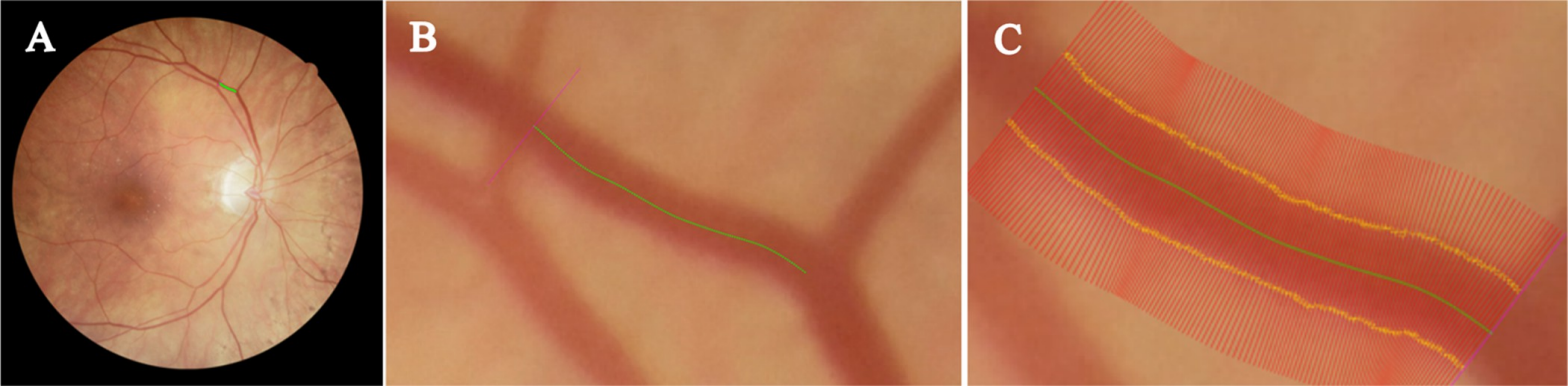
Vessel widths performed by human observer. (A) FOREVERP retinal image with vessel marked for width measurements (green). (B) Vessel segment showing a vessel centreline (green line). (C) Vessel segment with vessel widths (yellow crosses) marked by human observer.

An unsupervised approach was used for measuring vessel widths. This included creating centrelines (segmentation thinned) and edge points (zero-crossings of the second derivative), followed by measuring the distance between edge points orthogonal to the vessel centreline orientation (Fig 4) [27]. The test set consisted of 2,150 vessel profiles from 10 images of adequate quality. 961 profiles were from normal vessel segments without a strong central reflex, and with even illumination. 552 profiles showed a central reflex, and 637 profiles had low contrast or uneven illumination. Two human observers (JF, RAW) manually labelled the test set independently (Fig 5), and the mean of the two observers was used as the GT. To evaluate the agreement of measurements between QUARTZ and GT, a Bland-Altman plot was conducted.

### Arteriole and venule classification

Supervised learning was used for classifying vessels into arterioles or venules. This included the use of deep learning, specifically a 6-layered convolutional neural network [28]. A total of 100 images of adequate quality were randomly selected and divided into a training set, a validation set, and a test set consisting of 50, 15 and 35 images, respectively. Two human observers (JF, RAW) manually labelled 50 images each. Classification of vessels was evaluated on both pixels and vessel segments. A vessel segment refers to the part of a vessel between bifurcations and crossover points. The human observers used the following criteria for distinguishing between arterioles and venules [28,39]:

- Colour: Venules appear darker than arterioles.
- Diameter: The arterioles are thinner compared with adjacent venules.
- Central reflex: The central reflex is wider in arterioles compared with venules of comparable size [28,40].
- Branching: When labelling small vessels without colour differences or visible central reflexes, vessel branching was followed.

The sensitivity, specificity, and accuracy of the classification of arterioles and venules were examined for different probability thresholds; >0.5, >0.6, >0.7, >0.8, and >0.9.

### Optic disc localization

An unsupervised approach was used to determine the localization of the optic disc in the macular centre fundus images. This included the use of shade correction followed by the location of maximum intensity within a search region with constraints set. The test set comprised 300 images of adequate quality. One human observer manually labelled the images by marking the localisation of the optic disc.

## Results

### Automated image quality assessment

The ability of QUARTZ to detect low-quality images, evaluated on the 500 test set images, was calculated to have a sensitivity of 91.95% and a specificity of 95.64%. This equates to 80.40% of all images in the test set being labelled as of adequate quality (TN and FN) and of these, 98.26% were correctly labelled as of adequate quality (TN). When applying the automated algorithm to the full subset of 3,682 images, 80.55% (2,966 images) were labelled as adequate quality; with 95.17% of the participants having at least one image labelled as of adequate quality (Table 2). As these numbers include images from several years, they may overestimate the actual number of participants with an image of adequate quality. Evaluating images from one year (2021) showed consistency in image quality with 93.94% of the participants having at least one image labelled as adequate. The performances stated above equated to images being labelled as inadequate if the quality score was ≤ 0.48.

**Table 2:** Assessment of image quality of the FOREVERP dataset. Image quality evaluated as number (N) and percentage of participants with 0, 1, 2, 3, ≥1, ≥3 and images of adequate quality per participant in total and from one year (2021).

Images of adequate quality per participant	Total dataset		Data from the year 2021	
	Participants (N)	Participants (%)	Participants (N)	Participants (%)
**0**	55	4.83	69	6.06
**1**	81	7.11	118	10.37
**2**	619	54.35	829	72.85
**≥1**	1,084	95.17	1,069	93.94
**≥3**	384	33.71	122	10.72

### Vessel segmentation

The sensitivity of vessel segmentation performed by QUARTZ without pre/post-processing was 80.31% and 74.64% with pre/post-processing (Table 3). The specificity was 98.02% and 98.41% without and with pre/post-processing, respectively. Compared with the GT, the 2^nd^ human observer (JF) achieved a sensitivity of 75.07% and a specificity of 98.29%. Thus, the performance of QUARTZ was comparable to that achieved by the 2^nd^ human observer.

**Table 3:** Vessel segmentation of the FOREVERP dataset. Performance parameters of vessel segmentation listed for human observer and QUARTZ with and without pre/post processing compared to ground truth data. Performance evaluated in terms of sensitivity, specificity, and accuracy.

Method	Sensitivity	Specificity	Accuracy
2^nd^ Human observer	75.07%	98.29%	96.21%
QUARTZ (Without pre/post-processing)	80.31%	98.02%	96.44%
QUARTZ (With pre/post-processing)	74.64%	98.41%	96.28%

### Vessel width measurements

**Fig 6:**
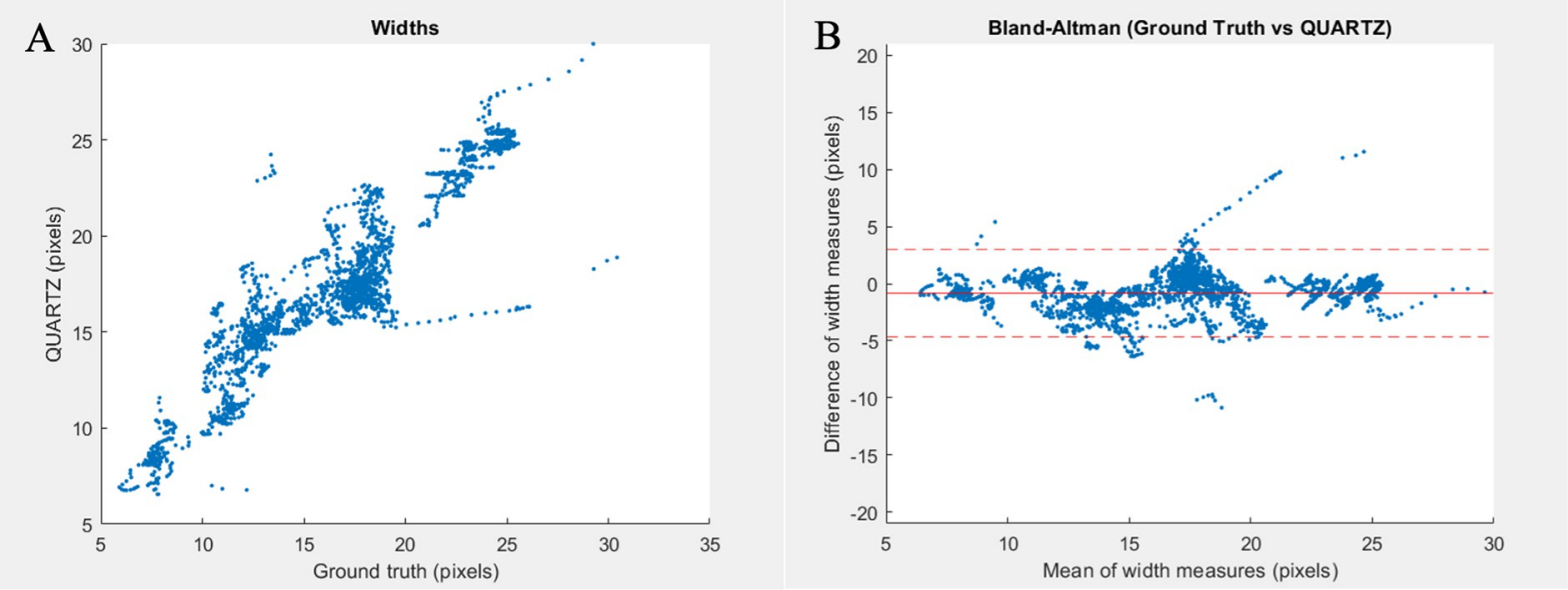
Vessel widths. (A) Measurements of vessel widths in pixels plotted for ground truth on x-axis against and QUARTZ on y-axis. (B) Bland-Altman plot of ground truth vs. QUARTZ with mean of width measures (pixels) on x-axis, differences of width measures (pixels) on y-axis, overall mean differences of width measurements (solid red line) with 95% CI (dashed red line).

In general, QUARTZ measured vessels as having a width greater than the GT (Table 4). The mean difference (± standard deviation) calculated as the GT minus QUARTZ varied from -1.40 (2.23) pixels (vessels with low contrast/uneven illumination) to -0.37 (1.91) pixels (normal vessel segments). The overall difference for all vessel profiles was -0.80 (1.96) pixels. The correlation coefficient between the GT and QUARTZ was 0.9111, demonstrated via a scatter plot in Fig 6. The differences between the GT and QUARTZ were overall stable across vessel widths showing minor linear patterns that might be vessel segment specific as visualised by the Bland-Altman plot (Fig 6). However, there appears to be little systematic error. A low variance in width measurements between the GT and QUARTZ ensures that the obtained width measurements are measured consistently. A low variance is thus more important compared with the absolute difference between the GT and QUARTZ [27].

**Table 4:** FOREVERP width measurements. Width measurements in pixels listed as mean (μ) + standard deviation (σ) for the following vessel segments: normal vessel segment, vessels with central reflex, vessels with low contrast/uneven illumination and an average of all vessel profiles measured by human observer 1, human observer 2, ground truth and QUARTZ. Also, the differences μ (σ) between ground truth and QUARTZ are listed for all vessel profiles addressed.

Vessel segment	Human observer 1		Human observer 2		Ground Truth (GT)		QUARTZ		Difference GT vs. QUARTZ	
	μ	σ	μ	σ	μ	σ	μ	σ	μ	σ
Normal vessel segments	16.33	4.24	15.87	5.12	16.10	4.50	16.47	4.34	-0.37	1.91
Vessels with central reflex	16.75	5.70	18.14	6.83	17.45	6.18	18.29	6.06	-0.85	1.44
Vessels with low contrast/uneven illumination	16.01	3.65	14.62	2.79	15.32	3.10	16.72	2.40	-1.40	2.23
All vessel profiles	16.34	4.52	16.08	5.26	16.21	4.73	17.01	4.49	-0.80	1.96

### Arteriole and venule classification

Since an arteriole is followed by an adjacent venule, the two classes (arteriole and venule) are approximately balanced, and therefore assessing accuracy is sufficient. The accuracy for the classification of arterioles and venules was evaluated with respect to per pixel and segment. The accuracy was 89.25% per pixel and 86.32% per segment for both arterioles and venules. Other performance measurements are provided in Table 5.

**Table 5:** Arteriole and venule classification performed by QUARTZ on the FOREVERP dataset. Performance evaluated in terms of sensitivity, specificity, and accuracy per pixels and per vessel segments.

Level	Measure	Arteriole	Venule
**Pixel**	Accuracy	89.25%	89.25%
	Sensitivity	90.23%	88.36%
	Specificity	88.36%	90.23%
**Segment**	Accuracy	86.32%	86.32%
	Sensitivity	88.64%	84.26%
	Specificity	84.26%	88.64%

By increasing the probability threshold for arteriole/venule classification, the sensitivity, specificity, and accuracy were improved. However, increasing the threshold and thereby improving the performance of the algorithm resulted in a loss of data (Table 6). Increasing the probability threshold to >0.8 more than halved the dataset. A threshold of >0.9 increased the sensitivity and accuracy to exceed 99%, but at the cost of approximately 2/3 of the dataset. A threshold of 0.8 has been chosen in previous epidemiological studies as an appropriate threshold ensuring both high data volume and performance of the algorithm [3].

**Table 6:** Arteriole and venule classification. Sensitivity of arteriole and venule classification and the arteriole/venule accuracy of the FOREVERP dataset reported for the following thresholds: >0.5, >0.6, >0.7, >0.8, >0.9. The fraction of data retained for every threshold is reported alongside the performance parameters.

Thresholds	Arteriole sensitivity	Venule sensitivity	A/V Accuracy	Fraction of data retained
**A>0.5, V>0.5**	88.64%	84.26%	86.32%	1
**A>0.6, V>0.6**	93.75%	91.38%	92.53%	0.82
**A>0.7, V>0.7**	96.48%	95.84%	96.16%	0.65
**A>0.8, V>0.8**	98.36%	98.59%	98.48%	0.49
**A>0.9, V>0.9**	99.29%	99.70%	99.51%	0.31

### Optic disc localization

Of the 300 randomly selected images, QUARTZ demonstrated a detection rate of 97.33% in terms of correctly identifying the location of the optic disc.

## Discussion

Overall, QUARTZ demonstrated high test performance in measuring retinal vessel width on the FOREVERP dataset, exhibiting good agreement with ground truth measures when compared with previously published data from UK Biobank [27]. It is of great importance to show similar test performance of QUARTZ in a different geographic population using a different image acquisition system, as it is a recognized challenge for automated retinal image analysis software to perform well on multiple datasets that vary in population and protocols [41]. Poor agreement between vasculometry measures using different software may limit the homogeneity of the associations between retinal vasculometry and disease across studies.

The ability to show high performance across different datasets is highly relevant as it ensures the validity of the measurements and thus provides high quality input for future epidemiological studies investigating associations between vessel morphology and risk factors for disease.

High image quality is crucial for the reliability of the other parameters evaluated by the algorithm. The image quality of the FOREVERP dataset exceeded the image quality of the UK Biobank dataset, with 80.55% (FOREVERP) compared with 71.53% (UK Biobank) of the images labelled as adequate [27]. There may be various reasons that cause reduced image quality, including technical parameters such as poor illumination, lens artefacts, defocus, or blur. Other participant or operator-related factors may affect image quality by causing obstructions or multiple falsely segmented non-vessel objects, such as the capture of eyelashes, visible choroid layer, exudates, drusen, haemorrhages, retinitis pigmentosa, retinal scars, asteroid hyalosis or inflammation of the optic disc [26,42]. The high quality of the FOREVERP dataset compared with the UK Biobank dataset may partly be explained by better trained retinal photographers. In UK Biobank, the personnel had basic training in fundus imaging [26], whereas the camera operators in the FOREVERP dataset were trained personnel supervised by optometrists.

The segmentation of retinal vessels is challenging due to their complex structure and heterogeneity in terms of shape, size, and intensity. Moreover, illumination and poor contrast may further complicate distinguishing small vessels from background noise [43]. In the search for new retinal vascular risk factors, it is more important to ensure that the algorithm correctly avoids non-vessel objects. A high specificity ensures, that the marked vessels are correctly identified. The specificity of the overall vessel segmentation was similar for the two datasets, whereas the sensitivity and accuracy of vessel segmentation were improved in the FOREVERP dataset compared with the UK Biobank dataset (Table 7). By applying pre/post-processing to the images, the specificity of the FOREVERP (98.41%) and UK Biobank dataset (98.88%) increased, at the cost of reduced sensitivity. Also, the performance parameters of arteriole and venule classification were improved in the FOREVERP dataset compared with UK Biobank. Exceptions were the specificity of arteriole classification/sensitivity of venule classification evaluated per segment (Table 7). With the consistently high levels of both sensitivity and specificity, there is potential for future epidemiological and/or clinical studies using the FOREVER dataset. In that case, pre/post-processing of the images would be preferable to increase the certainty of correctly segmented retinal vessels.

**Table 7:** The performance of QUARTZ on the two datasets FOREVERP and UK Biobank. Performance evaluated on the following parameters: Vessel segmentation, arteriole, and venule classification. Performance parameters are addressed as sensitivity, specificity, and accuracy. Vessel segmentation reported with and without pre/postprocessing. Arteriole and venule classification reported per pixel and per segment.

Dataset Parameter	FORVEVERP			UK Biobank			
	Sensitivity	Specificity	Accuracy	Sensitivity	Specificity	Accuracy	Comment
Vessel segmentation	80.31%	98.02%	96.44%	73.66%	98.14%	95.64%	without pre/post-processing
	74.64%	98.41%	96.28%	69.12%	98.88%	95.84%	with pre/post-processing
**Arteriole classification**	90.23 (A)	88.36 (A)	89.25 (A)	86.07 (A)	87.67 (A)	86.97(A)	level: pixel
	88.64 (A)	84.26 (A)	86.32 (A)	85.14 (A)	85.32 (A)	85.24 (A)	level: segment
**Venule classification**	88.36 (V)	90.23 (V)	89.25 (V)	87.67 (V)	86.07 (V)	86.97 (V)	level: pixel
	84.26 (V)	88.64 (V)	86.32 (V)	85.32 (V)	85.14 (V)	85.24 (V)	level: segment

In the FOREVERP dataset, the mean (± standard deviation) difference between the GT data and the width predicted by QUARTZ was -0.80 (1.96), showing that QUARTZ detected vessels as having a width larger than the GT.

In the UK Biobank dataset, the difference between the GT and QUARTZ was 0.70 (1.13) [27], showing that QUARTZ detected vessels as having a width smaller than the GT (Table 8). The width measurements are very stable within each dataset and the discrepancy between datasets is likely explained by the subjective nature of human observers. The larger image size of the FOREVERP dataset (1.75x of UK Biobank) may alter the perception of the vessel edges perceived by the human observers causing the differences in how they annotate vessel widths between the two datasets. The magnitude of the mean difference in both datasets is similar (4.9% FOREVERP and 6.4% UKBB) when assessed as a comparison to the mean vessel profile width.

The detection rate of localising the optic disc correctly was similar for the two datasets with a detection rate of 97.33% for the FOREVERP dataset compared with 97.60% for UK Biobank [27].

**Table 8:** Mean (μ) width with standard deviations (σ) of vessel segments reported for the two datasets; FOREVERP and UK Biobank. μ (σ) listed for the following vessel types: normal, central reflex, low contrast/uneven illumination, and an average of all vessel profiles. Additional category of normal and central reflex for UK Biobank only.

Vessel Type	FOREVERP						UK Biobank					
	Ground Truth (GT)		QUARTZ		Diff. GT vs. QUARTZ		Ground Truth (GT)		QUARTZ		Diff, GT vs. QUARTZ	
	μ	σ	μ	Σ	μ	σ	μ	σ	μ	σ	μ	σ
Normal	16.10	4.50	16.47	4.34	-0.37	1.91	10.46	1.26	10.00	1.60	0.46	0.94
Central reflex	17.45	6.18	18.29	6.06	-0.85	1.44	10.76	1.31	10.06	1.51	0.70	1.01
Low contrast/uneven illumination	15.32	3.10	16.72	2.40	-1.40	2.23	11.29	0.85	10.56	1.89	0.73	1.78
Normal and central reflex	-	-	-	-	-	-	11.09	0.97	10.18	1.23	0.91	0.85
All vessel profiles	16.21	4.73	17.01	4.49	-0.80	1.96	10.87	1.16	10.17	1.54	0.70	1.13

As shown in this paper, QUARTZ demonstrated a high performance across two different datasets. Whilst this demonstrates robustness, calibration and training on each dataset were required. Therefore, a future generic version of QUARTZ would be the next step, compatible across multiple retinal datasets with no or minimal calibration. To achieve a more universal system, further validation of QUARTZ in multi-ethnic populations would be necessary, as they may present alterations in retinal pigmentation and disease risk profiles compared to a Danish and British population. All the modules/components of this next version will be more heavily driven by AI, specifically deep learning. Such a system would make widespread research or adoption to predict the risk of disease based on fundus image analysis more feasible. An alternative approach to vessel morphometry is end-to-end AI, in which a single deep learning model directly maps the input images to the risk of disease [42].

A wider application of AI in ophthalmology is promising, as ophthalmology is highly dependent on imaging modalities for diagnosis as well as monitoring disease progression [44]. AI has made large strides in recent years due to the rise of deep learning, for example, to predict the risk of disease or identify pathology [36,45], such as “*IDx-DR”*, which has been approved by U.S Food and Drug Administration (FDA) to screen for diabetic retinopathy [46,47]. However, AI (particularly end-to-end) approaches have mainly been developed for research purposes as the method is challenging to implement in a clinical setting. This is partly due to the complexity of the analysis performed by the algorithm, which makes it difficult to assess how the input data relates to the final output data [44,48–50]. To visualize the parameters on which the algorithm has made its decisions, heatmaps can be developed thus increasing transparency [49]. Nevertheless, it remains a challenge for the future implementation of AI in healthcare that the algorithms are not based on medical reasoning [49,51].

AI algorithms can be trained with publicly available datasets such as DRIVE (Digital Retinal Images for Vessel Extraction) [52], STARE (Structured Analysis of the Retina) [53], HRF (High-Resolution Fundus Image Database) [54] and CHASE_DB1 (a subset of retinal images from the Child Heart and Health Study in England) [55] or larger datasets such as UK Biobank [56] and EPIC (European Prospective Investigation into Cancer and Nutrition) [57], available to researchers upon approval of access to the databases. High-quality data are important to ensure the reliability of an algorithm, and the training and test data should match the data from the clinical population of interest [7]. AI methods rely on access to representative and comprehensive training datasets [11,49], and approaches that use deep learning often require large datasets to avoid overfitting models. Although algorithms perform well on a public dataset, there is no guarantee for a high performance on the clinical data of interest, as the datasets may vary both in terms of the technical settings and the population being studied. If the publicly available datasets are not suitable for the clinical data of interest, researchers may need to create larger datasets from appropriate settings, as in the case with the FOREVER dataset. With linkage to data from the comprehensive national registries, the FOREVER dataset can in future be used for large-scale epidemiological research.

## Conclusion

In conclusion, QUARTZ exhibited high performance on a subset of 3,682 retinal images from the FOREVERP dataset. Compared with previously published data from the UK Biobank, QUARTZ’s performance on the FOREVERP dataset was at the same level or higher. QUARTZ has hereby shown robustness across datasets, enabling future research linking vessel morphometry with epidemiological data aiming to find novel biomarkers of disease.

## References

[1] Courtie E, Veenith T, Logan A, Denniston AK, Blanch RJ. Retinal blood flow in critical illness and systemic disease: a review. Annals of Intensive Care. 2020;10(1):152. doi: 10.1186/s13613-020-00768-3 33184724 PMC7661622

[2] London A, Benhar I, Schwartz M. The retina as a window to the brain—from eye research to CNS disorders. Nature Reviews Neurology. 2013;9(1):44–53. doi: 10.1038/nrneurol.2012.227 23165340

[3] Owen CG, Rudnicka AR, Welikala RA, Fraz MM, Barman SA, Luben R, et al. Retinal Vasculometry Associations with Cardiometabolic Risk Factors in the European Prospective Investigation of Cancer-Norfolk Study. Ophthalmology. 2019;126(1):96–106. Epub 2018/08/01. doi: 10.1016/j.ophtha.2018.07.022 . 30075201 PMC6302796

[4] Rudnicka AR, Owen CG, Welikala RA, Barman SA, Whincup PH, Strachan DP, et al. Retinal Vasculometry Associations With Glaucoma: Findings From the European Prospective Investigation of Cancer–Norfolk Eye Study. American Journal of Ophthalmology. 2020;220:140–51. doi: 10.1016/j.ajo.2020.07.027 32717267 PMC7706353

[5] Cheung CY, Ikram MK, Klein R, Wong TY. The clinical implications of recent studies on the structure and function of the retinal microvasculature in diabetes. Diabetologia. 2015;58(5):871–85. doi: 10.1007/s00125-015-3511-1 25669631

[6] Raj A, Tiwari AK, Martini MG. Fundus image quality assessment: survey, challenges, and future scope. IET Image Processing. 2019;13(8):1211–24. doi: 10.1049/iet-ipr.2018.6212

[7] Rashidi HH, Tran NK, Betts EV, Howell LP, Green R. Artificial Intelligence and Machine Learning in Pathology: The Present Landscape of Supervised Methods. Acad Pathol. 2019;6:2374289519873088. Epub 20190903. doi: 10.1177/2374289519873088 ; PubMed Central PMCID: PMC6727099. 31523704 PMC6727099

[8] Ahuja AS. The impact of artificial intelligence in medicine on the future role of the physician. PeerJ. 2019;7:e7702. doi: 10.7717/peerj.7702 31592346 PMC6779111

[9] Jones LD, Golan D, Hanna SA, Ramachandran M. Artificial intelligence, machine learning and the evolution of healthcare: A bright future or cause for concern? Bone Joint Res. 2018;7(3):223–5. doi: 10.1302/2046-3758.73.BJR-2017-0147.R1 . 29922439 PMC5987686

[10] Davenport T, Kalakota R. The potential for artificial intelligence in healthcare. Future Healthc J. 2019;6(2):94–8. doi: 10.7861/futurehosp.6-2-94 . 31363513 PMC6616181

[11] Ahmad BU, Kim JE, Rahimy E. Fundamentals of artificial intelligence for ophthalmologists. Curr Opin Ophthalmol. 2020;31(5):303–11. doi: 10.1097/ICU.0000000000000679 . 32740061

[12] De Fauw J, Ledsam JR, Romera-Paredes B, Nikolov S, Tomasev N, Blackwell S, et al. Clinically applicable deep learning for diagnosis and referral in retinal disease. Nat Med. 2018;24(9):1342–50. Epub 20180813. doi: 10.1038/s41591-018-0107-6 . 30104768

[13] Campbell JP, Ataer-Cansizoglu E, Bolon-Canedo V, Bozkurt A, Erdogmus D, Kalpathy-Cramer J, et al. Expert Diagnosis of Plus Disease in Retinopathy of Prematurity From Computer-Based Image Analysis. JAMA Ophthalmol. 2016;134(6):651–7. doi: 10.1001/jamaophthalmol.2016.0611 ; PubMed Central PMCID: PMC5201139. 27077667 PMC5201139

[14] Wong TY, Bressler NM. Artificial Intelligence With Deep Learning Technology Looks Into Diabetic Retinopathy Screening. Jama. 2016;316(22):2366–7. doi: 10.1001/jama.2016.17563 . 27898977

[15] Keel S, Li Z, Scheetz J, Robman L, Phung J, Makeyeva G, et al. Development and validation of a deep-learning algorithm for the detection of neovascular age-related macular degeneration from colour fundus photographs. Clin Exp Ophthalmol. 2019;47(8):1009–18. Epub 20190725. doi: 10.1111/ceo.13575 . 31215760

[16] Ting DSW, Cheung CY, Lim G, Tan GSW, Quang ND, Gan A, et al. Development and Validation of a Deep Learning System for Diabetic Retinopathy and Related Eye Diseases Using Retinal Images From Multiethnic Populations With Diabetes. Jama. 2017;318(22):2211–23. doi: 10.1001/jama.2017.18152 ; PubMed Central PMCID: PMC5820739. 29234807 PMC5820739

[17] Li Z, Guo C, Nie D, Lin D, Zhu Y, Chen C, et al. A deep learning system for identifying lattice degeneration and retinal breaks using ultra-widefield fundus images. Ann Transl Med. 2019;7(22):618. doi: 10.21037/atm.2019.11.28 ; PubMed Central PMCID: PMC6944533. 31930019 PMC6944533

[18] Lee CS, Baughman DM, Lee AY. Deep learning is effective for the classification of OCT images of normal versus Age-related Macular Degeneration. Ophthalmol Retina. 2017;1(4):322–7. Epub 20170213. doi: 10.1016/j.oret.2016.12.009 ; PubMed Central PMCID: PMC6347658. 30693348 PMC6347658

[19] Motozawa N, An G, Takagi S, Kitahata S, Mandai M, Hirami Y, et al. Optical Coherence Tomography-Based Deep-Learning Models for Classifying Normal and Age-Related Macular Degeneration and Exudative and Non-Exudative Age-Related Macular Degeneration Changes. Ophthalmol Ther. 2019;8(4):527–39. Epub 20190812. doi: 10.1007/s40123-019-00207-y ; PubMed Central PMCID: PMC6858411. 31407214 PMC6858411

[20] Gulshan V, Peng L, Coram M, Stumpe MC, Wu D, Narayanaswamy A, et al. Development and Validation of a Deep Learning Algorithm for Detection of Diabetic Retinopathy in Retinal Fundus Photographs. Jama. 2016;316(22):2402–10. doi: 10.1001/jama.2016.17216 . 27898976

[21] Poplin R, Varadarajan AV, Blumer K, Liu Y, McConnell MV, Corrado GS, et al. Prediction of cardiovascular risk factors from retinal fundus photographs via deep learning. Nature Biomedical Engineering. 2018;2(3):158–64. doi: 10.1038/s41551-018-0195-0 31015713

[22] Hajian-Tilaki K. Sample size estimation in epidemiologic studies. Caspian J Intern Med. 2011;2(4):289–98. . 24551434 PMC3895825

[23] Perez-Rovira A, MacGillivray T, Trucco E, Chin KS, Zutis K, Lupascu C, et al. VAMPIRE: Vessel assessment and measurement platform for images of the REtina. Annu Int Conf IEEE Eng Med Biol Soc. 2011;2011:3391–4. doi: 10.1109/IEMBS.2011.6090918 . 22255067

[24] Dong M, Yan BP, Liao JK, Lam YY, Yip GWK, Yu CM. Rho-kinase inhibition: A novel therapeutic target for the treatment of cardiovascular diseases. Drug Discovery Today. 2010;15(15–16):622–9. doi: 10.1016/j.drudis.2010.06.011 20601092 PMC3807099

[25] Fraz MM, Welikala RA, Rudnicka AR, Owen CG, Strachan DP, Barman SA. QUARTZ: Quantitative Analysis of Retinal Vessel Topology and size–An automated system for quantification of retinal vessels morphology. Expert Systems with Applications. 2015;42(20):7221–34. doi: 10.1016/j.eswa.2015.05.022

[26] Welikala RA, Fraz MM, Foster PJ, Whincup PH, Rudnicka AR, Owen CG, et al. Automated retinal image quality assessment on the UK Biobank dataset for epidemiological studies. Comput Biol Med. 2016;71:67–76. Epub 2016/02/20. doi: 10.1016/j.compbiomed.2016.01.027 . 26894596

[27] Welikala R, Fraz M, Habib M, Daniel-Tong S, Yates M, Foster P, et al., editors. Automated quantification of retinal vessel morphometry in the UK biobank cohort. 2017 Seventh International Conference on Image Processing Theory, Tools and Applications (IPTA); 2017.

[28] Welikala RA, Foster PJ, Whincup PH, Rudnicka AR, Owen CG, Strachan DP, et al. Automated arteriole and venule classification using deep learning for retinal images from the UK Biobank cohort. Comput Biol Med. 2017;90:23–32. Epub 20170908. doi: 10.1016/j.compbiomed.2017.09.005 . 28917120

[29] Chua SYL, Thomas D, Allen N, Lotery A, Desai P, Patel P, et al. Cohort profile: design and methods in the eye and vision consortium of UK Biobank. BMJ Open. 2019;9(2):e025077. doi: 10.1136/bmjopen-2018-025077 30796124 PMC6398663

[30] Owen CG, Rudnicka AR, Welikala RA, Fraz MM, Barman SA, Luben R, et al. Retinal Vasculometry Associations with Cardiometabolic Risk Factors in the European Prospective Investigation of Cancer-Norfolk Study. Ophthalmology. 2019;126(1):96–106. Epub 20180801. doi: 10.1016/j.ophtha.2018.07.022 ; PubMed Central PMCID: PMC6302796. 30075201 PMC6302796

[31] Rudnicka AR, Welikala RA, Barman SA, Foster PJ, Luben R, Hayat SA, et al. Artificial intelligence enabled retinal vasculometry for prediction of circulatory mortality, myocardial infarction and stroke. medRxiv. 2022:2022.05.16.22275133. doi: 10.1136/bjo-2022-321842 36195457 PMC9685715

[32] Tapp RJ, Owen CG, Barman SA, Welikala RA, Foster PJ, Whincup PH, et al. Retinal Vascular Tortuosity and Diameter Associations with Adiposity and Components of Body Composition. Obesity (Silver Spring). 2020;28(9):1750–60. Epub 20200729. doi: 10.1002/oby.22885 ; PubMed Central PMCID: PMC7116641. 32725961 PMC7116641

[33] Tapp RJ, Owen CG, Barman SA, Welikala RA, Foster PJ, Whincup PH, et al. Associations of Retinal Microvascular Diameters and Tortuosity With Blood Pressure and Arterial Stiffness: United Kingdom Biobank. Hypertension. 2019;74(6):1383–90. Epub 20191030. doi: 10.1161/HYPERTENSIONAHA.119.13752 ; PubMed Central PMCID: PMC7069386. 31661987 PMC7069386

[34] Thakoor KA, Koorathota SC, Hood DC, Sajda P. Robust and Interpretable Convolutional Neural Networks to Detect Glaucoma in Optical Coherence Tomography Images. IEEE Transactions on Biomedical Engineering. 2021;68(8):2456–66. doi: 10.1109/TBME.2020.3043215 33290209 PMC8397372

[35] Freiberg J, Rovelt J, Gazzard G, la Cour M, Kolko M. Finding Ophthalmic Risk and Evaluating the Value of Eye exams and their predictive Reliability (FOREVER)-A cohort study in a Danish high street optician setting: Design and methodology. Acta Ophthalmol. 2023. Epub 20230504. doi: 10.1111/aos.15693 . 37140185

[36] Ting DSW, Peng L, Varadarajan AV, Keane PA, Burlina PM, Chiang MF, et al. Deep learning in ophthalmology: The technical and clinical considerations. Prog Retin Eye Res. 2019;72:100759. Epub 20190429. doi: 10.1016/j.preteyeres.2019.04.003 . 31048019

[37] Ahsan MM, Luna SA, Siddique Z. Machine-Learning-Based Disease Diagnosis: A Comprehensive Review. Healthcare (Basel). 2022;10(3). Epub 20220315. doi: 10.3390/healthcare10030541 ; PubMed Central PMCID: PMC8950225. 35327018 PMC8950225

[38] Zhu W, Zeng NF, Wang N, editors. 1 Sensitivity, Specificity, Accuracy, Associated Confidence Interval and ROC Analysis with Practical SAS. NESUG Proceedings: Health Care and Life Sciences, Baltimore, Maryland. 2010.

[39] Hatami N, Goldbaum M. Automatic Identification of Retinal Arteries and Veins in Fundus Images using Local Binary Patterns. ArXiv. 2016; abs/1605.00763.

[40] Claudia K, Daniel K, Michelle Y, editors. Blood vessel classification into arteries and veins in retinal images. ProcSPIE; 2007.

[41] Trucco E, Ruggeri A, Karnowski T, Giancardo L, Chaum E, Hubschman JP, et al. Validating retinal fundus image analysis algorithms: issues and a proposal. Invest Ophthalmol Vis Sci. 2013;54(5):3546–59. Epub 20130501. doi: 10.1167/iovs.12-10347 ; PubMed Central PMCID: PMC4597487. 23794433 PMC4597487

[42] Mookiah MRK, Hogg S, MacGillivray TJ, Prathiba V, Pradeepa R, Mohan V, et al. A review of machine learning methods for retinal blood vessel segmentation and artery/vein classification. Medical Image Analysis. 2021;68:101905. 10.1016/j.media.2020.101905. doi: 10.1016/j.media.2020.101905 33385700

[43] Khan KB, Siddique MS, Ahmad M, Mazzara M. A Hybrid Unsupervised Approach for Retinal Vessel Segmentation. BioMed Research International. 2020;2020:8365783. doi: 10.1155/2020/8365783 33381585 PMC7749777

[44] Hanif AM, Beqiri S, Keane PA, Campbell JP. Applications of interpretability in deep learning models for ophthalmology. Curr Opin Ophthalmol. 2021;32(5):452–8. doi: 10.1097/ICU.0000000000000780 ; PubMed Central PMCID: PMC8373813. 34231530 PMC8373813

[45] Bora A, Balasubramanian S, Babenko B, Virmani S, Venugopalan S, Mitani A, et al. Predicting the risk of developing diabetic retinopathy using deep learning. Lancet Digit Health. 2021;3(1):e10–e9. Epub 20201126. doi: 10.1016/S2589-7500(20)30250-8 . 33735063

[46] FDA. De Novo Classification Request For IDx-DR 2018 [cited 2022 19.09]. Available from: https://www.accessdata.fda.gov/cdrh_docs/reviews/DEN180001.pdf.

[47] RELEASE FN. FDA permits marketing of artificial intelligence-based device to detect certain diabetes-related eye problems FDA; 2018 [cited 2022 21.02]. Available from: https://www.fda.gov/news-events/press-announcements/fda-permits-marketing-artificial-intelligence-based-device-detect-certain-diabetes-related-eye.

[48] Coyner AS, Campbell JP, Chiang MF. Demystifying the Jargon: The Bridge between Ophthalmology and Artificial Intelligence. Ophthalmology Retina. 2019;3(4):291–3. 10.1016/j.oret.2018.12.008. doi: 10.1016/j.oret.2018.12.008 31014678 PMC7874933

[49] Choi RY, Coyner AS, Kalpathy-Cramer J, Chiang MF, Campbell JP. Introduction to Machine Learning, Neural Networks, and Deep Learning. Translational Vision Science & Technology. 2020;9(2):14–. doi: 10.1167/tvst.9.2.14 32704420 PMC7347027

[50] Schmidt-Erfurth U, Sadeghipour A, Gerendas BS, Waldstein SM, Bogunović H. Artificial intelligence in retina. Progress in Retinal and Eye Research. 2018;67:1–29. 10.1016/j.preteyeres.2018.07.004. doi: 10.1016/j.preteyeres.2018.07.004 30076935

[51] Hayashi Y. The Right Direction Needed to Develop White-Box Deep Learning in Radiology, Pathology, and Ophthalmology: A Short Review. Front Robot AI. 2019;6:24. Epub 20190416. doi: 10.3389/frobt.2019.00024 ; PubMed Central PMCID: PMC7806076. 33501040 PMC7806076

[52] Staal J, Abramoff MD, Niemeijer M, Viergever MA, Ginneken Bv. Ridge-based vessel segmentation in color images of the retina. IEEE Transactions on Medical Imaging. 2004;23(4):501–9. doi: 10.1109/TMI.2004.825627 15084075

[53] Guo S. DPN: detail-preserving network with high resolution representation for efficient segmentation of retinal vessels. Journal of Ambient Intelligence and Humanized Computing. 2021:1–14. doi: 10.1007/s12652-021-03422-3

[54] Budai A, Bock R, Maier A, Hornegger J, Michelson G. Robust vessel segmentation in fundus images. Int J Biomed Imaging. 2013;2013:154860. Epub 20131212. doi: 10.1155/2013/154860 ; PubMed Central PMCID: PMC3876700. 24416040 PMC3876700

[55] Fraz MM, Remagnino P, Hoppe A, Uyyanonvara B, Rudnicka AR, Owen CG, et al. An ensemble classification-based approach applied to retinal blood vessel segmentation. IEEE Trans Biomed Eng. 2012;59(9):2538–48. Epub 20120622. doi: 10.1109/TBME.2012.2205687 . 22736688

[56] UK Biobank Eye and Vision Consortium [cited 2022 19.09]. Available from: https://www.ukbiobankeyeconsortium.org.uk/.

[57] Riboli E, Kaaks R. The EPIC Project: rationale and study design. European Prospective Investigation into Cancer and Nutrition. International Journal of Epidemiology. 1997;26(suppl_1):S6–S. doi: 10.1093/ije/26.suppl_1.s6 9126529

